# Nanoencapsulation of Maqui (*Aristotelia chilensis*) Extract in Chitosan–Tripolyphosphate and Chenopodin-Based Systems

**DOI:** 10.3390/antiox13030273

**Published:** 2024-02-23

**Authors:** Daniela Andrade, Francisca Maldonado-Bravo, Amador Alburquerque, Camilo Pérez, Alexander Gamboa, Nelson Caro, Mario Díaz-Dosque, Martin Gotelland, Lilian Abugoch, Cristian Tapia

**Affiliations:** 1 Department of Food Science and Chemical Technology, Faculty of Chemical Sciences and Pharmacy, University of Chile, Carlos Lorca Tobar 964, Independencia, Santiago 8380494, Chile; daniela.andrade@ug.uchile.cl (D.A.); francisca.maldonado@ug.uchile.cl (F.M.-B.); camilo.perez.r@ug.uchile.cl (C.P.); alexander.gamboa@usach.cl (A.G.);; 2 Department of Environmental Sciences, Faculty of Chemistry and Biology, University of Santiago de Chile, Santiago 9170022, Chile; 3 Austral Biotech Research Center, Santo Tomas University, Santiago 8320000, Chile; nelsoncarofu@santotomas.cl; 4 Institute of Dental Sciences, Faculty of Dentistry, University of Chile, Santiago 8330015, Chile; mrdiaz@uchile.cl; 5 Department of Nutrition, Faculty of Medicine, University of Chile, Santiago 8330015, Chile

**Keywords:** maqui extract standardisation, maqui nanoencapsulation, antioxidant, chitosan, chenopodin, alginate

## Abstract

Maqui berries contain a high percentage of anthocyanins with high antioxidant and anti-inflammatory capacity but that are unstable in the colonic site. Nanocarriers based on polysaccharides and/or proteins can protect against the degradation of anthocyanins. The aim of this study was the nanoencapsulation of maqui extract (ME) in chitosan–tripolyphosphate (CTPP-ME), chenopodin (CH-ME), and chenopodin–alginate (CHA-ME). A standardised ME was prepared and then encapsulated in the nanosystems. The physicochemical properties, encapsulation parameters, and the interactions of ME with the nanovehicles were characterised. The cyanidin-3-glucoside released and ORAC activity in phosphate buffer at pH 7.4 were evaluated. The content of ME was 8–9 mg of cyanidin-3-glucoside/g of extract. CTPP with ME at 3% obtained the highest encapsulation efficiency (EE = 91%), and no significant differences were observed in size (274–362 nm), PDI (0.5–0.7), and zeta potential (+34–+41 mV) when the concentration of ME changed from 1% to 5%. CH-ME was shown to be smaller (152 nm) than CTPP-ME, and CH-ME and CHA-ME showed lower EE (79% and 54%, respectively) than CTPP-ME. FT-IR revealed a stronger interaction of ME with CTPP-ME than with CH-ME. Both systems showed a significantly lower release than free ME, and the T50 value of CTPP-ME 3% (328 min) was higher than CH-ME (197 min). Both protected the ORAC activity of ME.

## 1. Introduction

Maqui (*Aristotelia chilensis*) is a small evergreen shrub that produces small round dark violet berries due to its high concentration of anthocyanin pigments, with delphinidin being the most abundant. It is considered one of the berries with the highest antioxidant content; its three main polyphenolic components are phenolic acids, flavonoids, and anthocyanins [1,2]. Within these compounds, anthocyanins are known for their high antioxidant, anti-inflammatory, neuroprotective, and anticancer capacities [3]. These compounds are unstable because they contain unsaturated double bonds and easily oxidisable groups; their stability is related to the number and position of the hydroxyl groups and the degree of glycosylation and methylation. The main external factors that help the degradation of anthocyanins are pH, temperature, and O_2_ concentration [4]. Regarding their stability in the human gastrointestinal tract, it is known that they are stable in the gastric fluid (pH 1.5–2 and that at the level of the jejunum (pH 7.4–8), anthocyanins, the glycolysed form of anthocyanidin, are absorbed in through glucose transporters. Anthocyanidins are passively absorbed after the action of hydrolytic enzymes. The colonic microbiota degrades that fraction of anthocyanins not absorbed in the small intestine. Bacteria beneficial for intestinal homeostasis, such as Bifidobacterium spp. and Lactobacillus spp, can degrade anthocyanins first via the cleavage of the glycosidic bond, followed by the cleavage of the anthocyanidin heterocycle and subsequent degradation to phloroglucinol derivatives, and benzoic acids [3].

Nanocarriers have been proposed to protect anthocyanin from unfavourable environmental conditions, such as pH and microbiota degradation, and reduce the interaction with other diet components to improve bioavailability. Polysaccharides have been used as an encapsulation material because they are not digestible by digestive enzymes in the upper gastrointestinal tract but are degraded by the intestinal microbiota, which allows for localised release in the colon and can additionally have prebiotic effects, favouring the growth of beneficial bacteria and suppressing the growth of pathogenic bacteria [5]. Chitosan has been one of the most widely studied polysaccharides as a vehicle for site-specific release in the colon due to its ability to protect bioactive compounds from the drastic conditions of the upper gastrointestinal tract and release the encapsulated active compounds in the colon through the hydrolysis of the glycosidic bonds of chitosan by the bacterial microbiota [6]. Chitosan with different deacetylation degrees and molecular weights shows differences in terms of IBD therapeutic ability. However, the current data described that these subtle differences do not affect the overall therapeutic effect [7].

In particular, chitosan–tripolyphosphate nanoparticles (CTPP), obtained by ionic gelation, have been described for the encapsulation of cyanidin-3-glucoside [8] and for aronia anthocyanins [9]. Another nanosystem based on chitosan is the one described by Ge et al. [6], which reports the encapsulation of blueberry anthocyanins via the ionic gelation of chitosan hydrochloride and carboxymethyl chitosan [10]. However, this nanocarrier obtained by ionic gelation has poor stability in acidic environments and uneasiness in encapsulating high molecular weight actives [11]. Additionally, it has high batch-to-batch particle size, size distribution, and surface charge variations. One solution will be the use of microfluidics as a procedure to fabricate CTPP with monodisperse size distribution and precisely controlled morphology [12]. CTPP loaded with proanthocyanidin was tested against zebra fish for the testing of genotoxic outcomes. No malformations were seen in the zebrafish embryo, and larvae were incubated with a nanoparticle solution at different concentrations (1.56, 3.12, 6.25, 12.5, and 25 μg/mL), representing their low harmfulness to the living fish [13]

To date, the nanoencapsulation of maqui extract (ME) in CTPP has not been reported in the literature, and it is of interest to compare the encapsulation efficiency of this extract to other systems already described based on the ionic gelation of chitosan.

It is important to highlight the stability that proteins (plant and animal) provide to anthocyanins and the possibility of using these alone or combined with polysaccharides as nanovehicles. According to Rashwan et al. [14], the hydrophobic region of the proteins can interact with the benzene ring present in the structure of the anthocyanins. Likewise, the carbonyl and amine groups of the proteins form hydrogen bonds with the hydrophilic zone of this polyphenol. It has been described that the addition of soy proteins to anthocyanins allows for the formation of a nanocomplex with higher stability [15]. Adding bovine serum albumin at a concentration of 0.15 mg/mL to a blueberry extract inhibits the degradation of anthocyanins, maintaining their antioxidant capacity [16]. The combined use of polysaccharides with proteins improves the encapsulation efficiency of anthocyanins. Ge et al. [17] reported an increase in the encapsulation efficiency of blueberry anthocyanins in the chitosan hydrochloride–carboxymethyl chitosan system by adding β-lactoglobulin [17].

In our group, we have studied the intermolecular interactions and the physicochemical and structural properties of soluble chenopodine-alginate (CHA) nanocomplexes. The main interactions were hydrophobic hydrogen bonds and weak electrostatic interactions. Changes were observed in the secondary and tertiary structure of chenopodin (CH), as was an increase in its thermal stability. The most significant stability was achieved at a ratio of chenopodin/alginate 1/4 [18]. 

This work seeks to evaluate the encapsulation of maqui extract. To do this, first, we aim to standardise a maqui extraction (ME) procedure according to its cyanidin-3-glucoside content and then encapsulate it in two nanosystems: chitosan–tripolyphosphate (CTPP-ME) and nanosystems based on chenopodin (CH-ME) and chenopodin-alginate (CHA-ME) and compare both systems according to their encapsulation efficiency and loading capacity. Additionally, the cyanidin-3-glucoside released and the protection of ORAC activity in a media that simulated the pH (7.4) of the colonic site were evaluated. 

## 2. Materials and Methods

### 2.1. Plant Materials

Quinoa flour: Organic quinoa seed flour was obtained from Sociedad Comercial and Agrícola Promauka Ltd., Paredones, VI Region, Chile. The flour was stored at −30 °C until use, with the following proximal composition: 11.3% of moisture, 11.2% of protein, 4.9% of fat; 0.555% of ashes, and 72.6% of carbohydrates [19] freeze-dried Maqui acquired from Isla Natura de Chiloé-Chile.

### 2.2. Chemical Reagents

The following were obtained from Sigma-Aldrich (Saint Louis, MO, USA): low-molecular-weight chitosan (LMW), 448,869, with a degree of 75–85% deacetylation and a viscosimetric weight of 269 kDa [17]; low viscosity sodium alginate, 71,238, from brown algae with an average molar mass of 162 kDa and with an M/G (mannuronic/glucuronic) ratio of 1.2 [19]; Bradford reagent B6916; BSA, 10,711,454,001; cyanidin-3-glucoside hydrochloride; HPLC grade acetonitrile; Tween 80; sodium tripolyphosphate; 2,2′-azobis [2-amidinopropane] dihydrochloride (AAPH); and fluorescein, 6-hydroxy-2,5,7,8-tetramethylchroman-2-carboxylic acid (Trolox). All other reagents used were of analytical grade.

### 2.3. Methodology

#### 2.3.1. Maqui Extract Standardization

*Obtaining ethanolic maqui extract*: Nine grams of lyophilised maqui powder was weighed in an amber bottle and dissolved in 90 mL of a mixture composed of acidified EtOH/H_2_O (0.1% HCl, *v*/*v*) at an 80:20 ratio (*v*/*v*). Subsequently, the mixture was homogenised with ULTRA TURRAX (T-25 IKA) at 12,000 rpm for 5 min. The homogenised sample was centrifuged (HermLe, model Z323K, Oststeinbek, Germany) at 3000 rpm for 7 min at 4 °C, and the supernatant was recovered and filtered at a pressure of 0.2 atm (20 kPa) with a 0.45 µm filter (Durapore, PVDF Millipore, Burlington, MA, USA). The filtered supernatant was rotary evaporated (Buchi, RE-120, Switzerland) in a balloon covered with aluminium foil for 40 min at 50 °C and 0.91 atm (92 kPa). The product obtained had a pH of 4.3–4.5 and was stored in the dark at 4 °C.

*Characterisation of maqui extract by HPLC*: The method described by Genskowsky et al. [20] was used to measure the concentration of anthocyanins. For sample preparation, 200 µL of fresh extract was added to an amber flask, and 1800 µL of 1% (*v*/*v*) acetic acid in water was stirred until a homogeneous mixture was achieved. Then, approximately 1.5 mL of sample was filtered with 0.45 µm syringe filters (MCE, FMC201030-ZX) and added to an amber vial for analysis on HPLC in triplicate.

Method conditions: The analysis of anthocyanins was carried out using Waters Alliance 2695 chromatograph equipment, with a Purospher RP-18 column measuring 4.6 × 250 mm with a particle size of 5 µm. Detection was carried out using a PDA 996 Waters photodiode array, with a scan between 200 and 700 nm. The working flow was 1 mL/min, with the injection volume being 50 µL with a mobile phase A of acetonitrile, a mobile phase B of 1% (*v*/*v*) acetic acid in water, and a linear gradient according to the time in minutes and mobile phase A/B ratio % as follows: 0 min, 0%A/100%B; 2 min 0%A/100%B; 15 min, 20%A/80%B; 20 min, 30%/70%; 30 min, 40%A/60%B; 37 min, 40%A/60%B; 38 min, 0%A/100%B; and 40 min, 0%A/100%B. A standard cyanidin-3-glucoside calibration curve (R^2^ = 0.99) was established from a stock solution of cyanidin-3-glucoside. An amount of 0.733 mg of standard was dissolved in 0.5 mL of 1% (*v*/*v*) acetic acid in water. Then, 0.2 mL of the stock was diluted in 2 mL of 1% (*v*/*v*) acetic acid in water, and, from this last dilution, aliquots of 0.1 were taken (0.2, 0.4, 0.5, and 1 mL), which were transferred to 5 mL amber flasks, and the volume of 1 mL was completed with 1% (*v*/*v*) acetic acid in water. The method was linear with a coefficient of correlation and determination greater than R^2^ = 0.9998. The variance test showed that the intercept was not significant at 0 (*p* = 0.49); therefore, it passed through the origin. The relative standard deviation was less than 2.0% over the entire range of analytical concentrations.

#### 2.3.2. Nanoencapsulation Methods

Preparation of chitosan–tripolyphosphate loaded with maqui extract (CTPP-ME): Firstly, a stock solution of 0.3% (*w*/*v*) chitosan in 1% (*v*/*v*) acetic acid was prepared. Three different formulations were made for CTPP. The details are shown in Table 1.

Subsequently, the chitosan solution with the extract was left to stir overnight. Afterwards, a 0.1% (*w*/*v*) TPP solution was prepared, and 40 mL of the chitosan–extract mixture was dripped onto 20 mL of TPP solution at a drip rate of 1.8 mL/min (KD Scientific, Holliston MA, model KDS200, USA). The mixture obtained was centrifuged for 30 min at 13,500 rpm at 14 °C (HermLe, model Z323K, Oststeinbek, Germany), recovering the supernatant in falcon tubes covered with aluminium to protect from light. Furthermore, the precipitate was suspended in 5 mL of water for subsequent measurement in HPLC.

##### Preparation of Nanosystems Based on Chenopodin

Obtaining defatted quinoa flour for the purpose of obtaining 11S globulin from quinoa (chenopodin): The quinoa flour was suspended in hexane at a 1:10 ratio. The suspension was maintained for 4 h with continuous stirring at approximately 4 °C. Subsequently, the flour was filtered under vacuum (9 × 10^−4^ atm, 0.091 kPa) with Whatman No. 1 paper and allowed to dry for one day at room temperature in a hood until the solvent was eliminated [19]. Obtaining quinoa protein extract: The defatted quinoa flour was suspended with a buffer of 0.5 M NaCl/0.05 M Tris-HCl pH 8.0 at a 1:10 ratio to prepare the protein extract. The suspension was shaken for 1 h and subsequently centrifuged at 13,500 rpm (HermLe, model Z323K, Oststeinbek, Germany) for 30 min at 15 °C. The extract was prefiltered with Whatman No. 1 paper [19].

Obtaining 11S globulin concentrate fraction from quinoa by diafiltration/ultrafiltration (DF/UF): We used the method described by Arazo et al. [18], with modifications. The SARTOFLOW^®^ Slice 200 Benchtop Crossflow System ultrafiltration equipment was used, and stabilised cellulose membranes of 30 and 100 kDa were used in series with a work area of 0.02 m^2^ (Sartocon^®^ Slice 200 Hydrosart^®^ Cassettes, Bohemia, NY, USA). The system and membranes were rinsed with a buffer (0.5 M NaCl/0.05 M Tris-HCl pH 8.0) before use to remove residual impurities. First, diafiltration was carried out with both membranes in series; 200 mL of the protein extract plus 200 mL of buffer were added, which passed through the system, obtaining 200 mL of diafiltered extract (retained). This procedure was repeated four times, adding 200 mL of buffer each time. Finally, the 200 mL of the final retentate was concentrated, leaving only the 100 kDa membrane until obtaining ⅕ the initial volume. The chenopodin extract obtained was frozen at −20 °C for 12 h and subsequently lyophilised (IshinBioBase, model FD5508, Yangju, Republic of Korea) at a temperature of −55 °C and vacuum of 7 × 10^−4^ atm (0.07 kPa) for approximately three days. The concentration of proteins was determined in the 11S globulin concentrate fraction using the Bradford method [21]. A calibration curve was performed by preparing a stock solution of BSA in distilated water (conductivity < 2.0 µScm^−1^) with a 10 mg/mL concentration. The absorbance was measured in a spectrophotometer at a length of 595 nm, and the results were expressed in mg of soluble protein per mL of extract. Based on what was described by Intiquilla et al. (unpublished data), two different formulations were tested, one with the formation of a complex between chenopodin and alginate (CHA-ME) and another with chenopodin (CH-ME). The formulations are described in Table 2. For both formulations, the pH of the maqui extract was adjusted to 5.5 (1M HCl).

For the CHA-ME formulation, chenopodin was dissolved in 11 mL (11.5 g) of maqui extract, allowing the plate to stir for 60 min or until a homogeneous solution was achieved. The alginate was dissolved in the other 11 mL of extract and stirred for approximately 60 min. Then, both solutions were combined and stirred for another 30 min. After this time, the Tween 80 surfactant was added, and the solution was stirred for one hour. The mixture was taken to a sonicator (Q Sonica, Q700, Newtown, CT, USA) equipped with a 1.27 cm diameter probe, which was operated at a frequency of 20 kHz, with a power of 700 W with 25% amplitude and pulses (on-time/off-time) of 10 s on/15 s off. The solution was sonicated for 10 min in an ice bath, ensuring its temperature did not exceed 30 °C. Subsequently, the mixture was centrifuged at 13,500 rpm (HermLe, model Z323K, Germany) for 30 min at 14 °C. The supernatant was recovered, which was stored in the dark at 4 °C, and the pellet was resuspended in 5 mL of Milli-Q water for subsequent HPLC analysis.

Chenopodin was dissolved in the extract and stirred for 60 min for the CH-ME formulation. After that time, Tween 80 was added and left to stir for another 60 min. Then, the treatment was the same as for the CHA-ME formulation.

#### 2.3.3. Characterisation of Nanosystems

*Hydrodynamic size, PDI, and zeta potential* were determined for the nanocomplexes and nanoemulsions using the Zetasizer Nano ZS-20 equipment (Malvern Instrument, Malvern, UK). A total of 1 mL of the sample was placed in a capillary cuvette. The QTPP nanogels were previously diluted in water at a 1:1 ratio (sample: water). However, the chenopodin-based nanosystem (CHA-ME and CH-ME) samples were diluted in water at a 1:10 ratio.

*Determination of anthocyanin concentration by HPLC*: To prepare the sample, 1 mL of each fresh sample was taken and placed in a 10 mL volumetric amber flask, 5 mL of water was added, and the mixtures were sonicated (Sigma-Aldrich, model 2510EDTH, Branson, MO, USA) for 30 min. Subsequently, 5 mL of acetonitrile was added to the flask, and the mixture was sonicated again for 30 min. In the case of the precipitate of centrifugation, it was suspended in water, transferred to an amber flask, and sonicated for 30 min. Then, 5 mL of acetonitrile was added and sonicated for 30 min.

The sample solutions were previously filtered through 0.22 µm syringe filters (MCE, FMC201030-ZX, Honolulu, HI, USA) and transferred to their respective amber HPLC vials to be injected in triplicate.

The method and calibration curve conditions were the same as the maqui extract characterisation by HPLC. Identification was carried out by comparison of retention times and chromatograms with cyanidin-3-glucoside standard and maqui extract.

Encapsulation efficiency (*EE*) (Equation (1)) and loading capacity (*LC*) (Equation (2)):

(1) EE (%)=An−Ap/Ai

where:

An: mg of anthocyanins (expressed as mg equivalents of cyanidin-3-glucoside) contained in the nanosystem.

Ap: mg of anthocyanins (expressed as mg equivalents of cyanidin-3-glucoside) contained in the centrifugation precipitate of the nanosystems.

Ai: mg of anthocyanins (expressed as mg equivalents of cyanidin-3-glucoside) used to obtain the nanosystem.

(2) LC(%)=An/TM ×100

where

*An*: mg of anthocyanins (expressed as mg equivalents of cyanidin-3-glucoside) contained in the nanosystem.

*TM*: total mass of nanosystem components.

*Fourier transform infrared (ATR-FTIR) spectroscopy:* Infrared spectroscopy spectra were obtained from freeze-dried samples using a spectrometer (Thermo Scientific Nicolet 8700, Waltham, MA, USA) with attenuated total reflectance (ATR) in the 4000 and 450 cm^−1^ spectral range, averaging 50 scans with a resolution of 4 cm^−1^.

#### 2.3.4. In Vitro Release Study of Nanosystems in Phosphate Buffer at pH 7.4

In a stainless-steel basket, 10 mg of lyophilised product (ME, CTPP-ME 3%, CH-ME) was placed between 0.45 µm membranes (MCE). The basket was placed inside a 100 mL amber bottle, to which 50 mL of phosphate buffer of 0.05 M KH_2_PO_4_ composition adjusted with 5 M NaOH to pH 7.4 was added. The bottle was shaken in a shaker (PolyScience model, Niles, IL, USA) at 37 ± 0.5 °C and 50 ± 5 rpm. At times of 5, 40, 80, 120, 160, 200, and 240 min, samples were taken with a syringe, replenished in volume with buffer, and filtered through 0.45 μm membranes (MCE) into 1.5 mL amber vials. The test was carried out in triplicate.

*Quantitative determination of anthocyanins was performed by HPLC* using a Waters Alliance 2695 model Separations Module with PDA 996 diode array detector and a Symmetry C8 column measuring 3.9 × 150 nm with a pore size of 5 µm. The mobile phase comprised acetonitrile (solvent C) and 4.5% formic acid (solvent D). A 20-min gradient elution was performed with a flow rate of 1.00 mL/min and an injection volume of 100 μL with an initial composition of 100% D for 2 min, followed by a 30:70 (C:D) ratio for 10 min, 60:40 (C:D) for 4 min, and 100% D for 4 min. The anthocyanins were quantified by recognising the peak areas corresponding to wavelengths of 524 nm. The area value with 100% release was quantified from 10 mg of lyophilised sample of maqui extract in 50 mL of 1% glacial acetic acid by applying vortexing at pulses of 4 min with a 1-min pause for 15 min. The same quantification conditions were carried out for CTPP-ME 3% and CH-ME samples with some modifications. For CTPP-ME 3%, sonication was performed for 90 min, and vortexing was maintained constant by applying every 30 min for 5 min. For CH-ME, sonication was performed for 30 min, and vortexing was applied every 10 min for 5 min. The anthocyanin concentration was obtained from a calibration curve using cyanidin–3–glycoside as a standard.

*Oxygen radical scavenging capacity (ORAC) analysis*: We followed the procedure according to Huang [22], with some modifications. Briefly, a 75 mM phosphate buffer (PBS) (pH 7.4) and a calibration curve of 96 mM Trolox were prepared. In triplicate, successive dilutions of Trolox were made taking 10, 20, 30, 40, 50, 60, and 80 μL to make up to 1000 μL of PBS. The samples to be analysed, in triplicate, were diluted 1:50, 1:100, 1:200, 1:300, 1:400, 1:500, and 1:1000. The evaluation was carried out in a 96-well, flat black microplate, to which 150 μL of fluorescein (25 μM) and 25 μL of each dilution of the Trolox calibration curve were added per well. A blank sample was included by adding 25 μL PBS. Likewise, 25 μL of each sample was incorporated into the flatback plate in triplicate. Subsequently, the plate was incubated for 25 min at 37 °C, and 25 μL of the AAPH rad0.5ical at 96 mM was added to each well to start the reaction. Immediately, the fluorescence intensity was measured every 2 min for 120 min, with an excitation wavelength of 485 nm and emission 520 nm at 37 °C. Fluorescence measurements were normalised to the blank curve. From the normalised curves, the area under the fluorescence decay curve (*AUC*) was calculated according to Equation (3)

(3) AUC=0.5+f1/f0+…fi/f0 

where ƒ0 is the initial fluorescence reading at 0 min and ƒ*i* is the fluorescence reading at time *i*.

The net *AUC* corresponding to a sample was calculated according to Equation (4)

(4) Net AUC=antioxidantAUC−blank AUC

The slope of the equation was used to calculate the ORAC-FL value using the Trolox curve obtained for each trial. The final ORAC values were expressed in μmol Trolox equivalents/100 g of sample.

The data were evaluated via multifactorial ANOVA analysis using STATGRAPHICS Centurion XV software, version II (Statpoint Technologies, Inc., Warrenton, VA, USA). The responses used were the ORAC values, expressed in μmol Trolox equivalents/100 g of sample, concerning the following factors: formulation (ME code 1; CH-ME code 2; and CTPP-ME code 3) and time of contact with buffer phosphate (5, 40, 80, 120, 160, 200, and 240 min).

## 3. Results and Discussion

### 3.1. Standardisation of Maqui Extract

The anthocyanin concentration results for batches of maqui extract are shown in Table 3.

Batches 1, 2, and 4 did not present significant differences (*p* > 0.05). Batch 3 had the lowest concentration of anthocyanins and high variability between injections. Therefore, in the standardised maqui extract, the concentration of cyanidin-3-glucoside /mL was in the range of 4.11 to 4.35 mg, which corresponds to 8.62 to 9.13 mg cyanidin-3-glucoside per gram of lyophilised extract.

Table 4 compares the maqui extract concentration obtained in this study with published data in the literature. According to Table 4, the concentration found in this study was similar to concentrations indicated in the literature, that is, between 8 and 9 mg of cyanidin-3-glucoside/g of extract, not including the higher concentration obtained by Genskowsky et al. [20]. Regarding the components present in the extracts, Genskowsky et al. [20] found that the predominant anthocyanins were those derived from delphinidin, representing 66.42%, with delphinidin-3-glucoside as the most significant component. The cyanidin-3-glucoside obtained represented almost 3% of the anthocyanins, with a concentration of 1.24 ± 0.02 g/kg. In contrast, Tanaka et al. [23] found 0.9% cyanidin-3-glucoside in maqui extract using aqueous ethanol as a solvent. On the other hand, Rojo et al. [24] found that the raw maqui extract had 21% cyanidin-3-glucoside.

In aqueous solutions, anthocyanins undergo structural rearrangements due to changes in pH in four molecular structures: the flavylium cation, the quinoidal base, the carbinol, and the chalcone. Anthocyanins are more stable in acidic solutions; at a pH between 1 and 3, they primarily have the structure of the flavylium cation, and at pH above 4, the anthocyanins take the form of carbinol and chalcone, the latter of which can undergo chemical degradation that produces phenolic acids [5,25]. The stability of anthocyanins has been studied at different pH levels. The greatest stability of these bioactive compounds, up to 60 days stored at 10 °C, was found at a pH range of 1 to 3. It was also found that at pH levels between 5 and 7, anthocyanins suffered degradation over time [26]. According to Cavalcanti et al. [27], the increase in pH causes a rapid loss of the proton-producing forms of quinonoidal bases (QB) with blue or violet colours. At the same time, the hydration of the flavylium cation (FC) occurs, generating the carbinol or pseudobase (PB) that slowly reaches equilibrium with the chalcone (CH). Given that the pH of the extract obtained was between 4.3 and 4.5, the structure of the anthocyanins present would be in an equilibrium between the carbinol pseudo base and the chalcone form.

**Table 4:** Comparison of different concentrations of maqui extracts.

Source	Mixtures of Solvents Used in the Extraction	mg Cyanidin-3-Glucoside/g Extract	Reference
Maqui Isla Natura-Chiloé-Chile	acidified (0.1% HCl) ethanol/water (80:20)	8.62–9.13	This research
Maqui Cañete-Chile	acidified (0.1% HCl) methanol/water (80:20)	22.58	[20]
Maqui Fundación Chile	70%methanol, cleaned up through AmberliteXAD-7	8.30	[24]
Maqui Paredones- Chile	acidified methanol (0.1% HCl)	8.82	[28]

### 3.2. Obtaining and Characterisation of Nanosystems

#### 3.2.1. Chitosan–Tripolyphosphate Loaded with Maqui Extract (CTPP-ME)

Table 5 shows the characterisation by dynamic light scattering (DLS) of the formulations described in Table 2.

The CTPP-ME did not show significant differences in size, PdI, and zeta potential when increasing the concentration from 1% to 3%. These results indicate that increasing the extract concentration does not produce particle aggregation. Table 6 compares the results obtained in this work with other results published regarding anthocyanin encapsulations using the ionic gelation of chitosan with tripolyphosphate. The results obtained in this work agree with those obtained by Liu et al. [8] since a particle size of 288 nm was obtained, which is very close to the value of 298 nm obtained by CTPP-ME 3%. Regarding the zeta potential, the value is also very similar. In contrast, Wang et al. [9] obtained a smaller particle size and polydispersity index but a zeta potential similar to that obtained in CTPP-ME 3%.

#### 3.2.2. Nanosystems Based on Chenopodin–Alginate (CHA) and Chenopodin (CH) Loaded with Maqui Extract (ME)

##### Obtaining 11S Globulin (Chenopodin, CH) from Quinoa Flour

Chenopodin was obtained through the diafiltration/ultrafiltration process. The protein concentration measured in duplicate using the Bradford method was 56.3–57.1 mg/mL of protein in the 11S globulin concentrate fraction. In the study by Arazo et al. [19], electrophoresis was performed on the protein extract obtained under reducing and non-reducing conditions to identify quinoa 11S globulin. For this, the researchers took different samples in the diafiltration (DF) process and a sample of the final concentrate. Under non-reducing conditions, there was a band between 46 and 58 kDa; under reducing conditions, there were bands between 30–35 kDa and 18–21 kDa, corresponding to the acidic and basic subunits of the globulin. Similar results were obtained by Arazo [29], describing a band between 47 and 63 kDa in non-reducing conditions, corresponding to the 11S globulin, and bands of 31 and 25 kDa in reducing conditions, corresponding to the acidic and basic subunits, respectively.

Table 7 shows the characterisation by DLS of the formulations described in Table 2. CH-ME had a significantly smaller size than CHA-ME, which was expected due to the size of alginate (169 kDa) compared to chenopodin (54 kDa), and a lower negative zeta potential. In the case of the polydispersity index (PdI), it would be expected that CHA-ME would be higher due to the formation of the nanocomplex. The type of core–shell of the nanostructure of CHA would explain this result. According to the results of Arazo [29], the nanocomplex of CHA, at the same ratio used in this work, without encapsulation of bioactive agent, had a z-average of 119.47 ± 7.69 nm, PdI of 0.46 ± 0.04, and zeta potential of −39.62 ± 4.14 mV. The CH had a z-average of 176.15 ± 4.88 nm, zeta potential of −15.65 ± 1.63 mV, and PdI of 0.19 ± 0.01. These results indicate that the interaction with alginate generates a higher-order structure due to hydrophobic interactions, hydrogen bonds, and weak electrostatic interactions between CH and alginate [18]. CH-ME and CHA-ME had a negative zeta potential due to the fabrication pH (5.5). At this pH, chenopodin was above its isoelectric point (4.5), and alginate was fully ionised (pKa 3–3.5) [18,30].

##### Encapsulation Efficiency (EE) and Load Capacity (LC) in the Nanosystems

The results obtained for the CTPP-ME are shown in Table 8.

CTPP-ME 3% obtained the highest encapsulation efficiency, and the highest loading of the ME was obtained by CTPP-ME 5%. Liu et al. [8] obtained an efficiency of 44.90% and a bioactive loading of 4.30% for the encapsulation of cyanidin through ionic gelation, with a ratio between chitosan and cyanidin-3-glucoside of 1:10. Sharif et al. [31] conducted a review on anthocyanin nano- and microencapsulation methods, where it was found that blueberry anthocyanins were encapsulated through ionic gelation, using chitosan hydrochloride and carboxymethyl chitosan, with an efficiency of 44%. However, when β-lactoglobulin was added to the chitosan hydrochloride and carboxymethyl chitosan mixture, the encapsulation efficiency improved to 69.4%. These results demonstrate that it is possible to encapsulate anthocyanins with efficiencies over 90% in CTPP 3%.

Table 9 shows the encapsulation parameters of maqui extract in nanosystems based on chenopodin (CH-ME and CHA-ME).

CH-ME showed a higher efficiency and loading capacity of maqui extract, 79% and 3.7%, respectively, than CHA-ME. Compare these results with other results published in the literature: Šaponjac et al. [32] encapsulated cherry anthocyanins by freeze-drying using whey and soy proteins, obtaining 94.90 and 90.10% efficiencies, respectively; Rashid et al. [33] encapsulated pomegranate anthocyanins with maltodextrin and whey protein isolate, achieving an encapsulation efficiency of 88.2%; and Arroyo-Maya and McClements [34] encapsulated an extract rich in anthocyanins with whey protein isolate and pectin (2:1 ratio, *w*/*w*), obtaining an efficiency of 52%.

**Figure 1:**
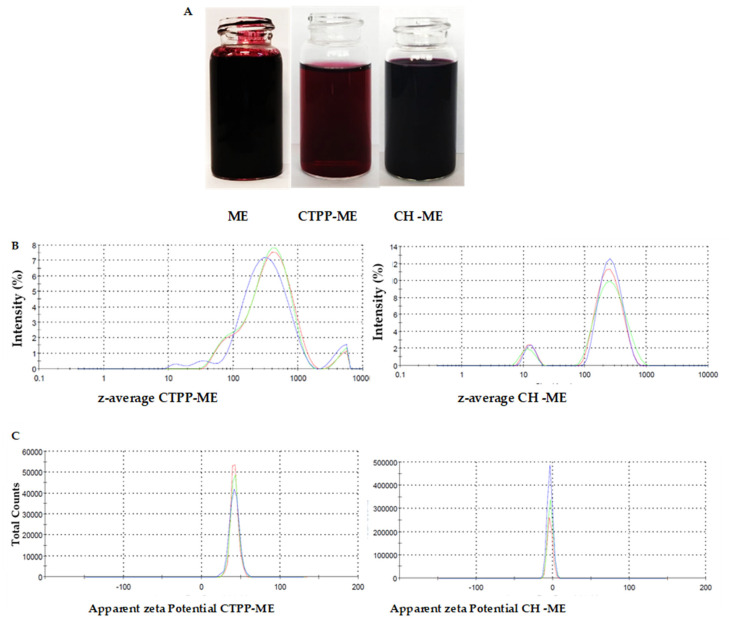
Maqui extract (ME) and nanoencapsulated in CTPP (CTPP−ME) and chenopodin (CH −ME) (**A**) Visual appearance of ME, CTPP−ME, and CH−ME. (**B**) Size distribution of ME, CTPP −ME, and CH−ME. (**C**) Zeta potential of CTPP-ME and CH-ME.

Figure 1 shows the highly coloured ME at 3% and nanovehicles loaded with ME (CTPP-ME 3% and CH-ME). CTPP-ME 3% was slightly less coloured compared to CH-ME (A). Both nanosystems showed a bimodal size distribution where CSTTP-ME 3% had a mean size distribution higher than CH-ME (B). CTPP-ME 3% had a positive and higher absolute zeta potential value compared to CH-ME, which showed a low and negative zeta potential.

##### FT-IR Analysis of Maqui Extract (ME), Chitosan (CS), Maqui Extract Nanoencapsulated in CTPP (CTPP-ME), Chenopodin (CH), and Chenopodin Maqui Extract (CH-ME)

**Figure 2:**
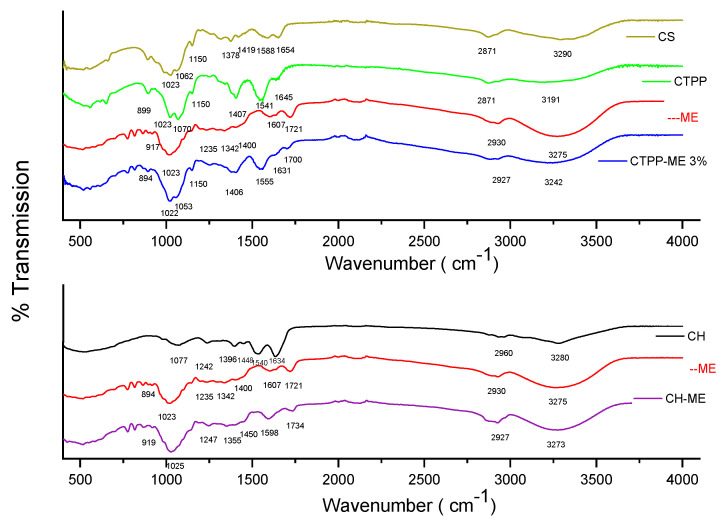
FT−IR of maqui extract (ME), chitosan (CS), maqui extract nanoencapsulated in CTPP (CTPP−ME), chenopodin (CH), and chenopodin maqui extract (CH−ME).

It can be seen from Figure 2 that CS exhibits a prominent signal at 3290 cm^−1^, indicative of O–H stretching and intramolecular hydrogen bonds. Additionally, absorption bands around 2873 cm^−1^ are associated with C–H symmetric and asymmetric stretching, respectively. The presence of residual N-acetyl groups is confirmed by bands at approximately 1654 cm^−1^ (C=O stretching of amide I) and 1588 cm^−1^, reflecting the characteristic peak of protonated amino groups (-NH_3_^+^) [35]. The bands at 1378 cm^−1^ are attributed to C-H vibrations in the chitosan structure [36], as well as the third band characteristic of typical N-acetyl groups, potentially overlapping with other bands. Bands at approximately 1410 cm^−1^ and 1378 cm^−1^ affirm the CH_2_ bending in the CH_2_OH groups and CH_3_ symmetrical deformations of residual N-acetyl groups, respectively. Furthermore, the absorption band at 1150 cm^−1^ corresponds to the asymmetric stretching of the C–O–C bond, while bands at 1062 cm^−1^ and 1023 cm^−1^ are attributed to C–O stretching. These bands align with spectra reported in previous studies on chitosan [18].

CTPP signals at 3191 cm^−1^ correspond to OH stretching. The absorption bands at approximately 2915, 2871 cm^−1^ are attributed to C–H symmetric stretching. In addition to the band at 1645 cm^−1^ due to C=O stretching of amide I, there is a shift in the N–H bending of the charged amino group at 1541 cm^−1^ [35]; this confirms the involvement of the –NH3^+^ group of CS in the electrostatic interactions with the phosphate group of the TPP. Additionally, a band at 1407 cm^−1^ is attributed to bending vibrations of –OH and –CH, and at 1020 cm^−1^ to C–O stretching [37]. The absorption band at 1150 cm^−1^ can be attributed to the asymmetric stretching of the C–O–C bond. The bands at 1070 and 1023 cm^−1^ correspond to C–O stretching –CH [34], which at 899 cm^−1^ presents the stretching vibration characteristic of P–O–P of TPP.

ME shows the broadband at 3275 cm^−1^ assigned to sugar vibrations and phenol O-H groups [38,39]. The peaks at 1721 cm^−1^ and 1607 cm^−1^ are attributed to the C=O and C=C groups for aromatic rings, respectively [38]. The bands at 1400 cm^−1^ and 1342 cm^−1^ correspond to the C–O deformation of phenols; the absorbance at 1234 cm^−1^ is associated with the stretching of the pyran rings that is characteristic of flavonoid compounds [40]. The absorbance at 1023 cm^−1^ can be attributed to the stretching vibration of the C–O–C esters, while the absorption band observed at 895 cm^−1^ indicates the presence of aromatic rings.

CH exhibits a sharp signal at 3280 and 2960 cm^−1^, which is assigned to O-H and N-H group stretching, respectively. The peaks at 1634 cm^−1^ correspond to C-O stretching (amide I), while those at 1540 cm^−1^ represent N-H bending (amide II). Additionally, the peak at 1448 cm^−1^ indicates C-O stretching vibration. The presence of a peak at 1396 cm^−1^ is characteristic of free amino acids as well as the terminal COO- groups of protein molecules. Moreover, the peak at 1242 cm^−1^ is associated with C-H bending vibration [41], and that at 1077 cm^−1^ is attributed to various groups undergoing out-of-plane C-H bending.

The CTPP-ME 3% nanosystem manifests four distinctive regions that elucidate the nature of interaction between their components:

(a) The CTPP-ME 3% reveals a signal associated with OH, detected at 3242 cm^−1^, demonstrating a deviation from the OH signal in CTPP at 3191 cm^−1^. This deviation implies a significant interaction between CTPP and ME. (b) CTPP-ME 3% signals emerge at 1700 cm^−1^ and 1631 cm^−1^, indicating the formation of an amide bond between the amide I of chitosan and a C=O group of ME. (c) The signal at 1555 cm^−1^ in CTPP-ME 3% reflects the degree of protonation of the amine group of chitosan (NH3^+^), with a signal at 1598 cm^−1^ [35]. These results imply that the interaction of CTPP with ME would also be an electrostatic interaction. (d) Notably, the peak at 1062 cm^−1^ associated with -CH-OH in chitosan cyclic alcohols shifts to 1053 cm^−1^ in CTPP-ME 3%, indicating an interaction.

In the CH-ME mixture, the signal at 3273 cm^−1^ associated with the OH group exhibits a minimal shift compared to ME at 3280 cm^−1^, implying a relatively minor interaction. Furthermore, a residual signal at 1734 cm^−1^ is observed in CH-ME, originating from ME at 1721 cm^−1^. Similarly, the signal at 1598 cm^−1^ suggests a low level of interaction between the two compounds. This consistent trend extends to the remaining signals as their shifts are not significant when compared to the pure products, ME and CH. These findings indicate that the interactions of ME with CTPP are more pronounced than those observed in CH-ME.

#### 3.2.3. In Vitro Release of Maqui Extract from CTPP-ME 3% and CH-ME in PBS 1X Buffer at pH 7.4

**Figure 3:**
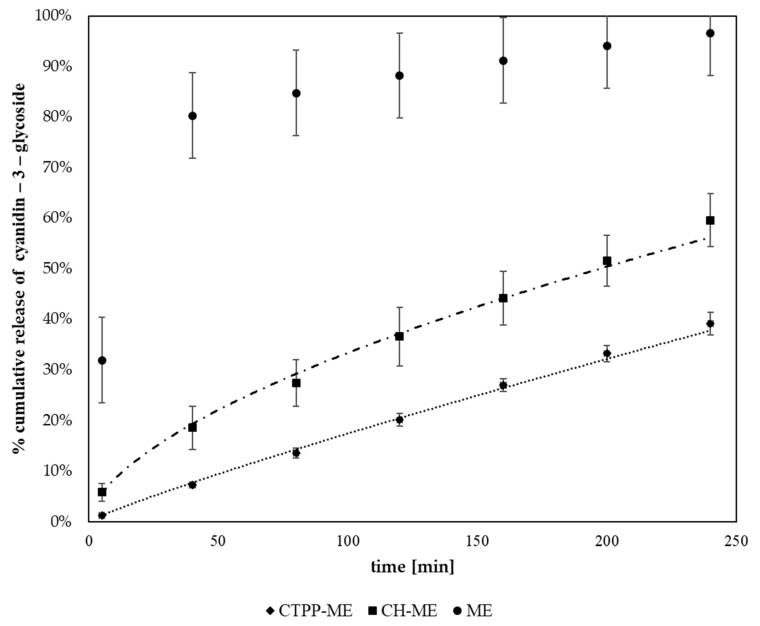
Profile release cyanidin-3-glycoside of maqui extract (ME), chitosan (CS), maqui extract nanoencapsulated in CTPP (CTPP−ME), chenopodin (CH), and chenopodin maqui extract (CH−ME).

The in vitro release of maqui extract measured as cyanidin-3-glycoside was developed in an orbital shaker using phosphate buffer saline buffer at pH 7.4 as the dissolution medium. Figure 3 shows the profile release of free maqui extract and maqui extract nanoencapsulated in CTPP-ME 3% and CH-ME. Both nanoencapsulated systems show a significantly lower release (*p* < 0.05) than free maqui extract, 60% for CH-ME and 39% for CTPP-ME 3%, compared to 97% for ME at 240 min. The in vitro dissolution data of multi-particulate, anti-inflammatory drug delivery systems based on polysaccharides and proteins are fitted mainly to the Korsmeyer–Peppas model because this describes drug release from a polymeric system considering non-Fickian mechanisms [42]. In this study, the dissolution data for CH-ME (K = 0.0215; *n* = 0.5954 R^2^ = 0.997) and CTPP-ME 3% (K = 0.003; *n* = 0.8833 R^2^ = 0.9987) fitted well to the Korsmeyer–Peppas model. The T50 values estimated from this model show 197.3 min for CH-ME and 327.6 min for CTPP-ME 3%. Previously, we described the viscoelastic behaviour of CTPP loaded with prednisolone and inulin in phosphate buffer at pH 7.4. CTPP showed values of G′ > G″ typical of a true gel, and both moduli showed flat mechanical spectra with slight frequency dependence under low-strain conditions [43]. Additionally, according to the results of FT-IR, CTPP-ME 3% showed a higher hydrogen bond interaction than CH-ME and a robust amide bond between the amide I of chitosan and a C=O group of the extract. In addition, the peak at 1062 cm^−1^ associated with -CH-OH in chitosan cyclic alcohols shifted to 1053 cm^−1^ in CTPP-ME 3%, indicating an interaction not observed in CH-ME. Thus, the mechanical behaviour of the nanovehicle and the high interaction of maqui extract with chitosan-TPP would explain the higher T50 value for CTPP-maqui extract at 3% compared to chenopodin-maqui extract.

#### 3.2.4. ORAC Activity of Free and Nanoencapsulated Maqui Extract

The ANOVA analysis was used to break down the variability of the ORAC value, expressed as µmol ET/100 g, into contributions due to two main factors: formulations (ME, CH-ME, and CTPP-ME 3%) and contact time (5, 40, 80, 120, 160, 200, and 240 min) with phosphate buffer at pH 7.4, which acts as a medium that simulates the pH of the colonic medium (pH 7.4). The interaction between the main factors (formulations × time) was also considered. The *p* values of the formulations (*p* = 0.0000), contact time (*p* = 0.0001), and the interaction between these factors (*p* = 0.0000) indicate that all of them had a statistically significant effect on the ORAC value with a 95.0% confidence level. The multiple range test per formulation shows that the nanoencapsulated formulations (CH-ME: 7624 ± 568 µmol ET/100 g; CTPP-ME 3%: 7063 ± 568 µmol ET/100 g) did not present significant differences between them in the mean value during the study period, but both were significantly lower than the ME (ME: 13,505 ± 568 µmol ET/100 g), which is reasonable considering the low loading capacity (LC) of both nanosystems (see Table 8 and Table 9).

**Figure 4:**
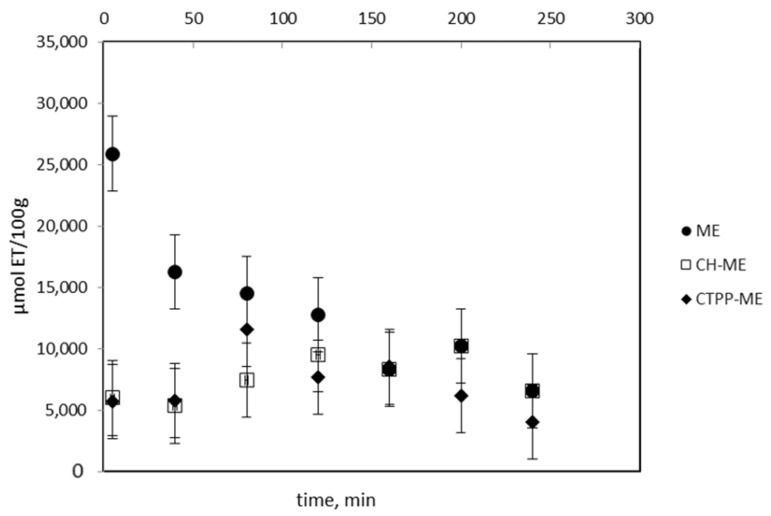
ORAC activity of free ME and ME nanoencapsulated in buffer phosphate pH 7.4.

Figure 4 shows the mean ORAC value with its 95% confidence interval during the study period for each formulation. In the case of ME, a significant drop in the ORAC value was observed between 5 min (25,898 ± 3032 µmol Et/100 g) and 40 min (16,278 ± 3032 µmol Et/100 g). After this time, a decreasing trend in the ORAC average value was shown, but it was not significant due to the high variability between samples. On the other hand, for the encapsulated formulations (CH-ME and CTPP-ME 3%), no significant differences were observed between them in their ORAC value over time. These results indicate that both nanoencapsulation systems would protect the antioxidant capacity of the maqui extract in a medium that simulates the pH of the colonic medium (pH 7.4). The effectiveness of CH-ME at protecting the ORAC activity of ME could be explained by previous reports that show that the globulin–anthocyanin complexes have greater DPPH and ABTS radical scavenging in comparison with albumin, prolamin, and glutelin due to the more flexible and unfolded structure of globulins [44].

## 4. Conclusions

Batch production under a standardised maqui extract procedure shows a low variability in the concentration of cyanidin-3-glucoside, which allowed a more controlled process of nanoencapsulation; the content of anthocyanidin was similar to the majority of published data in the literature.

The increase in maqui extract concentration from 1% to 3% does not modify the size and charge of CTPP-ME, which indicates that the increase in extract concentration does not generate particle aggregation. In the case of the chenopodin system, the complexation with alginate (CHA-ME) produced a significant increase in size compared to chenopodin alone (CH-ME). However, PDI did not change significantly, which would be explained by CHA’s core–shell nanostructure due to hydrophobic interactions, hydrogen bonds, and weak electrostatic interactions between CH and A.

Regarding the encapsulation efficiency, CTPP-ME 3% was the best. This result demonstrates that it is possible to encapsulate maqui extract with an efficiency of over 90%. Chenopodin systems were less efficient in encapsulating the extract. The interactions between the maqui extract and both nanocarriers evaluated by FT-IR showed that the interactions of ME with CTPP are more pronounced than those observed in CH-ME. This high interaction of ME with CTPP would explain the higher T50 value for CTPP-ME 3% compared to CH-ME obtained from in vitro release studies in PBS 1X buffer at pH 7.4. The ORAC activity of ME in this media was preserved when ME was encapsulated in both nanocarriers. This kind of nanosystem has the limitations of high batch-to-batch particle size, size distribution, and surface charge variations. One of the solutions proposed in the literature is using microfluidics to obtain nanoparticles with monodisperse size distribution and precisely controlled morphology.

## Figures and Tables

**Table 1:** Formulations of chitosan–tripolyphosphate loaded with maqui extract (CTPP-ME).

Formulations	Maqui Extract (mL)	Chitosan 0.3% (p/v) (mL)	TPP 0.1% (p/v) (mL)
CTPP-ME 1%	0.5	49.5	20
CTPP-ME 3%	1.5	48.5	20
CTPP-ME 5%	2.5	47.5	20

**Table 2:** Formulations of chenopodin–alginate (CHA) and chenopodin (CH) loaded with maqui extract (ME).

Components	CHA-ME	CH-ME
Maqui Extract (g)	23	23
Chenopodin (g)	0.72	1.2
Alginate (g)	0.48	-
Tween 80 (g)	0.8	0.8
Total (g)	25	25

**Table 3:** Anthocyanin concentration results for batches of maqui extract.

Batch	Anthocyanin Concentration (mg Equivalent Cyanidin-3-Glucoside/mL)
1	4.35 ± 0.01 ^a^
2	4.34 ± 0.01 ^a^
3	3.92 ± 0.28 ^b^
4	4.11 ± 0.03 ^ab^

Different letter means significant differences (*p* < 0.05).

**Table 5:** Particle size, PdI, and Zeta potential for CTPP-ME.

Formula	z-Average (nm)	PdI	Zeta Potential (mV)
CTPP-ME 1%	361.52 ^a^ ± 111.62	0.61 ^b^ ± 0.18	38.04 ^d^ ± 15.35
CTPP-ME 3%	273.67 ^a^ ± 20.85	0.67 ^b^ ± 0.23	41.00 ^d^ ± 1.87
CTPP-ME 5%	321.93 ^a^ ± 79.27	0.51 ^bc^ ± 0.06	33.68 ^d^ ± 11.42

Values are expressed as the average ± SE of the samples. Different letters indicate significant differences between rows, *p* < 0.05; *n* = 9.

**Table 6:** Comparison between CTPP-ME 3% and CTPP loaded with anthocyanin, as described in the literature.

Source of Anthocyanin	Anthocyanin Concentration (mg/mL)	Chitosan	Chitosan/ TPP Ratio	z-Average nm	PdI	Zeta Potential mV	Reference
Aronia Melanocarpa Extract	3	Sigma MMW 310 kDa	1/3 p/v	196.5	0.03	42.7	[9]
Cyanidin-3- glucoside	0.1	Beijing KehuajingweiScientific Co.Ltd. Chitosan oligosaccharides	5/1 p/p	288	-	30	[8]
Maqui extract	4.2	Sigma LMW 269 kDa	2/1 *v*/*v*	273.7–361.5	0.51–0.68	33.7–41.0	This research

**Table 7:** Characterisation results of nanosystems based on chenopodin.

Nanosystem	z-Average (nm)	PdI	Zeta Potential (mV)
CHA-ME	589.47 ^a^ ± 75.08	0.57 ^b^ ± 0.05	−8.73 ^c^ ± 0.15
CH-ME	152.23 ^b^ ± 10.32	0.57 ^b^ ± 0.04	−5.24 ^d^ ± 0.10

Values are expressed as the average ± SE of the samples. Different letters indicate significant differences between rows, *p* < 0.05; *n* = 6.

**Table 8:** Encapsulation efficiency (%) and loading capacity (%) of maqui extract in CTPP.

Formulations	EE (%)	LC (%)
CTPP-ME 1%	47.860 ^a^ ± 6.24	0.755 ^d^ ± 0.05
CTPP-ME 3%	90.984 ^b^ ± 2.50	3.248 ^e^ ± 0.06
CTPP-ME 5%	68.261 ^c^ ± 2.09	4.227 ^f^ ± 0.12

Values are expressed as the average ± SE of the samples. Different letters indicate significant differences between rows, *p* < 0.05; *n* = 9.

**Table 9:** Encapsulation efficiency (%) and loading capacity (%) of maqui extract in CHA and CH.

Formulation	EE (%)	LC (%)
CH-ME	78.92 ^a^ ± 2.35	3.67 ^c^ ± 0.10
CHA-ME	53.78 ^b^ ± 4.26	2.40 ^d^ ± 0.03

Values are expressed as the average ± SE of the samples. Different letters indicate significant differences between rows, *p* < 0.05; *n* = 6.

## Data Availability

Data are contained within the article.
